# Which transgender and gender diverse groups benefit most from E-health? Subgroup analyses of the randomized controlled trial i^2^TransHealth in Germany

**DOI:** 10.1080/15532739.2025.2465710

**Published:** 2025-02-22

**Authors:** Janis Renner, Peer Briken, Arne Dekker, Lea Pregartbauer, Antonia Zapf, Susanne Sehner, Amra Pepić, Matthias Augustin, Timo O. Nieder

**Affiliations:** a Institute for Sex Research, Sexual Medicine and Forensic Psychiatry, University Medical Center Hamburg-Eppendorf, Hamburg, Germany; b Institute of Medical Biometry and Epidemiology, University Medical Center Hamburg-Eppendorf, Hamburg, Germany; c Institute for Health Services Research in Dermatology and Nursing (IVDP), University Medical Center Hamburg-Eppendorf, Hamburg, Germany

**Keywords:** Effectiveness, E-health, mental health, RCT, telehealth, transgender healthcare

## Abstract

**Background:**

Transgender and gender diverse (TGD) people, particularly those living in underserved remote areas, often face barriers to accessing primary and specialty healthcare services due to geographic isolation and the scarcity of trained professionals in these regions. While e-health interventions have gained prominence in TGD-informed healthcare, no research to date has examined their subgroup-specific effectiveness in addressing the challenges faced by TGD people.

**Aim:**

This study aimed to investigate the responsiveness of various subgroups within the TGD population in Germany to the i^2^TransHealth e-health intervention, shedding light on its potential to mitigate disparities in healthcare access and outcomes through digital solutions such as video consultations and chat conversations.

**Methods:**

In this secondary analysis of a randomized controlled trial (RCT) conducted in Hamburg, Germany, involving 174 TGD study participants from four northern federal states of the country, we assessed the impact of the i^2^TransHealth e-health intervention on psychological distress.

**Results:**

Subgroup analyses revealed that i^2^TransHealth demonstrated effectiveness for TGD individuals overall, with higher age and education levels being associated with greater reductions in psychological distress. Clinically meaningful differences in treatment effectiveness were not observed in other subgroup variables, including residence size.

**Conclusion:**

E-health interventions like i^2^TransHealth offer promise in addressing the specific challenges faced by TGD people, particularly those in underserved remote areas. To enhance inclusivity and equity, interventions should consider the diverse needs of this population, emphasizing the importance of tailored approaches that accommodate varying educational backgrounds and geographic contexts.

The trial was pre-registered at Clinicaltrials.gov (NCT04290286).

## Introduction

Accessing specialized healthcare services poses persistent challenges for transgender and gender diverse (TGD) people (Hughto et al., 2015; Ross et al., 2023), with barriers including the shortage of healthcare professionals (HCPs) who are sufficiently trained to meet the needs of TGD people (de Vries et al., 2020; Lykens et al., 2018; Safer et al., 2016; Stryker et al., 2022; van Heesewijk et al., 2022). Frequently, TGD people find either no or insufficient specialized healthcare services (Burgwal & Motmans, 2020; European Union Agency for Fundamental Rights, 2020, 2024; James et al., 2017), particularly in remote areas, necessitating reliance on centralized settings in metropolitan areas (Koehler, Strauss, et al., 2021; Renner et al., 2021). This geographical disparity is associated with long travel distances, resulting costs, and missed time from their studies or careers (Hughto et al., 2016; Renner et al., 2021; Whitehead et al., 2016), especially in parts of the world with heightened TGD stigma. Vulnerable and multiply marginalized TGD people face exacerbated difficulties in accessing appropriate TGD-informed healthcare to this pronounced stigma (Burgwal & Motmans, 2020; Falck & Bränström, 2023; Smiley et al., 2017). Under these circumstances, some TGD people either struggle to access TGD-informed healthcare or avoid healthcare services altogether (European Union Agency for Fundamental Rights, 2020, 2024). Given these obstacles, this study evaluates the suitability of an e-health intervention for different subgroups within the TGD community in northern Germany.

The last decades have seen a growing trend toward digitalization in healthcare practices, such that across many disciplines, HCPs have offered digital care to their patients (e.g., telehealth, chat conversations, email, online health courses) (Andersson, 2018; Barak et al., 2009; Enam et al., 2018; Sood et al., 2007). While a variety of definitions of digital or virtual care have been proposed, this article adopts the e-health definition by Eysenbach (2001), as explored in a systematic review on e-health in the context of TGD-informed healthcare (Renner et al., 2022). Recent developments in TGD-informed healthcare have heightened the need for e-health, thus WPATH SOC 8 also mentions efforts to decentralize TGD-informed healthcare and make use of e-health services for improved access for TGD people (Coleman et al., 2022). In light of recent events related to the COVID-19 pandemic, it is becoming extremely difficult to ignore the usefulness of e-health services (Doraiswamy et al., 2020; Garfan et al., 2021; Hincapié et al., 2020; Khoshrounejad et al., 2021). As a result of actions taken to contain the COVID-19 pandemic, many healthcare facilities, including TGD-informed healthcare, around the world shifted much of their treatment to an online format (Hamnvik et al., 2022; Lock et al., 2022; Mintz et al., 2022).

There is a growing body of literature that recognizes the importance of e-health for TGD people (Radix et al., 2022; Renner et al., 2022; Smalley et al., 2022; Stoehr et al., 2022; Wong et al., 2022). However, much of the research up to now has been descriptive in nature, highlighting TGD people’s acceptance of e-health services as a new treatment modality (Hertling et al., 2021; Russell et al., 2022; Sequeira et al., 2021; Silva et al., 2021). Further complicating matters, e-health studies published to date often include TGD people in a broad LGBTQIA+ sample, which in some cases limits TGD-specific subgroup analyses due to case numbers (Renner et al., 2022). Although previous research tends to support the potential effectiveness of e-health services for TGD people, its generalizability remains limited due to differences in access to and use of technology. Scientific monitoring and effectiveness testing of e-health treatments in TGD-informed healthcare have been largely overlooked, so that unresolved issues persist in many countries, e.g., technological difficulties or insurance coverage problems (Peine et al., 2020). Addressing these concerns requires systematic evidence evaluations to reduce reservations and enhance understanding of how e-health services specifically contribute to improvements in TGD-informed healthcare.

This article aims to fill this knowledge gap in e-health services for TGD people, focusing on the i^2^TransHealth intervention. i^2^TransHealth combines video consultations and 1:1 chat conversations with mental health professionals from the University Medical Center Hamburg-Eppendorf, alongside optional crisis interventions by a trained local physician network in northern Germany if needed, aiming to provide accessible and cost-effective care (Nieder et al., 2022). A randomized controlled trial (RCT) was conducted to evaluate the effectiveness of location-independent video consultations and 1:1 chat conversations, seeking to reduce barriers and inconveniences associated with face-to-face treatment.

This present article explores subgroups of TGD study participants of i^2^TransHealth, analyzing descriptive sample characteristics and treatment response patterns. TGD-informed healthcare is interdisciplinary and highly specialized, so a transfer of its treatment services to the digital world should also be tailored for different target groups (Renner et al., 2022). Despite the importance of professional treatment provision tailored to the individual, there remains a paucity of evidence on how to deliver e-health care in a way that is appropriate for all. In Germany, the S3 guidelines (Deutsche Gesellschaft für Sexualforschung, 2018) recommend a comprehensive diagnosis of gender incongruence or gender dysphoria by a mental health professional in order to develop individual solutions with TGD people based on shared decision-making and informed consent, and to refer them for transition-related treatments such as hormone therapy or gender-affirming surgery (Nieder & Strauß, 2019). This requires interdisciplinary collaboration between mental healthcare, endocrinology, surgery, and other medical (e.g., hair removal) and non-medical (e.g., voice and speech therapy) specialties. While the interfaces between these disciplines are most often centrally coordinated in metropolitan areas in Germany, there are gaps in decentralized care in more remote regions (Koehler, Strauss, et al., 2021). Thus, e-health services could help TGD people in these areas to have better access to interdisciplinary healthcare in Germany.

This article has two primary aims: firstly, to show the results of explorative secondary analyses in terms of the Brief Symptom Inventory-18 (BSI-18) Global Severity Index (GSI), as part of the RCT; and secondly, to identify points for a requirements profile of future e-health services for TGD people. The primary analysis of the i^2^TransHealth study revealed significant and clinically relevant differences between the intervention and the waitlist control group and indicated the effectiveness of i^2^TransHealth (Nieder et al., 2024), motivating our investigation into how specific subgroups respond to treatment. We analyzed sociodemographic and intersectional variables of the baseline measurement (T0) to characterize relevant subgroup characteristics.

Tailoring e-health services to the health needs and preferences of TGD groups could potentially provide patient-centered and culturally competent care, thereby reducing access barriers, enhancing healthcare utilization and optimizing resource allocation. Our exploratory secondary analysis of i^2^TransHealth represents a pioneering effort in e-health research in TGD-informed healthcare, providing starting points for future in-depth analyses.

## Methods

### Recruitment

Recruitment for the i^2^TransHealth e-health intervention took place between the first participant’s inclusion in May 2020 to the final follow-up of the last participant in May 2022, following a study protocol (Nieder et al., 2022) with pre-registration on Clinicaltrials.gov (NCT04290286). Prospective study participants could contact us *via* our study website (www.i2transhealth.de), the institute secretariat, or our local physician network to request an initial interview in person at the study center. In each of these ways, they were informed about i^2^TransHealth and the associated participation in an RCT. In addition to these avenues of outreach, we actively recruited in local TGD communities in advance, distributed project flyers, gave interviews, and published articles in German journals. There was no monetary compensation for those interested in the study. However, i^2^TransHealth offered the prospect of care, which was the incentive to participate in the study.

### Participants

**Figure 1.**
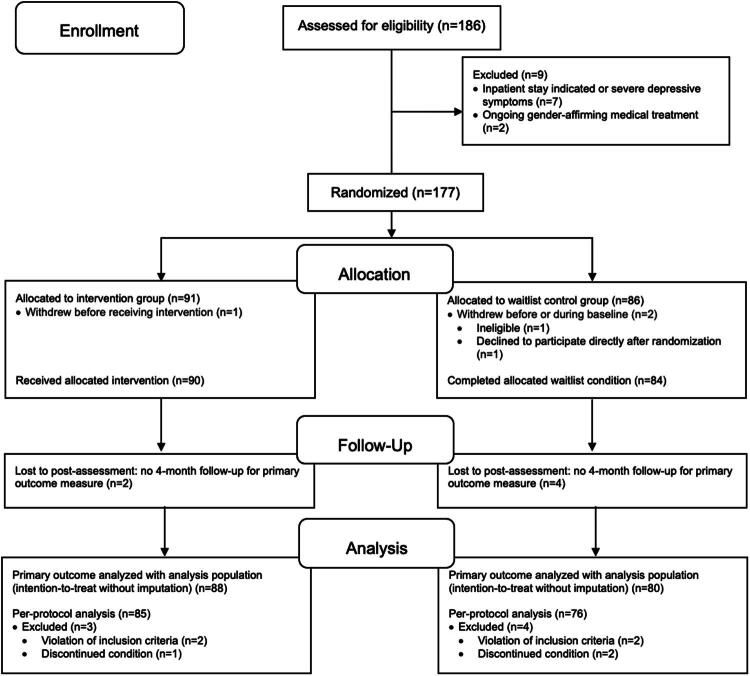
CONSORT flow diagram. Of the 177 randomized study participants, 3 people dropped out at very short notice a few days after study entry and had not completed any baseline questionnaires, so the intention-to-treat (ITT) population consisted of 174 people. Of these, 168 people with a complete follow-up T1 assessment were included in the analysis population. 161 people were included in the per-protocol analysis (PPA), exclusion in the PPA due to violation of inclusion criteria referred to initiation of gender-affirming medical treatments parallel to the study (total *n* = 5, *n* = 2 in the intervention group and *n* = 3 in the control group, with one person in the control group already dropped out of the PPA, resulting in *n* = 2 for the control group in the CONSORT flow diagram). An interim discontinued condition and a condition restarted before T1 during the study also resulted in exclusion from PPA (i.e., short-term withdrawal from the study and reentry into the study during the intervention or waiting period). This figure is adapted from the original version published in The Lancet Digital Health (Nieder et al., 2024) and has been modified for this publication.

The study included adult TGD people from northern Germany who lived at least 50 ­kilometers from the Hamburg metropolitan area, covering the four northern German federal states of Bremen, Lower Saxony, Mecklenburg-Western Pomerania, and Schleswig-Holstein. We prioritized substantial distance from specialized care over population density, acknowledging that even participants residing in cities faced inadequate access to specialized care. All potentially eligible participants underwent a personal initial interview and, if study fit, could be enrolled in the RCT. Detailed inclusion and exclusion criteria, along with the selection process, were outlined previously (Nieder et al., 2022). A total of 174 TGD people participated: 90 in the intervention group (referred to as service users) and 84 in the waitlist control group (Figure 1). Participants gave verbal and written informed consent prior to study participation.

### Design

This article presents explorative secondary analyses of quantitative data from the i^2^TransHealth e-health intervention study. The responses of all participants who provided complete data within the respective subgroup at both survey dates were used for the analyses. The aim of the single-masked RCT was to evaluate the effectiveness of the i^2^TransHealth intervention compared to a waitlist control group. Participants completed questionnaires at baseline before randomization (T0) and after four months of study participation (T1). Those who were allocated to the intervention group were able to start the intervention immediately, whereas the waitlist control group were only able to take up the intervention offer after four months. Post-study, both groups had the option to continue or start treatment.

The primary objective of our study was to determine whether the i^2^TransHealth intervention prevents worsening of psychological distress (BSI-18 GSI) in the intervention group compared to a waitlist control, with the null hypothesis of no significant difference in change in distress from baseline (T0) to follow-up (T1) between the RCT groups. To assess potential beneficiaries of i^2^TransHealth, we performed subgroup analyses related to the primary outcome, the change from baseline at four months in BSI-18 GSI, considering the following subgroups: assigned sex at birth (male, female), age (<25 years, ≥25 years), gender identity (trans man/trans masculine, trans woman/trans feminine, non-binary), residence size (rural/small-town: <5000 to 20,000; non-rural: >20,000 according to the German Federal Office for Building and Regional Planning), contact frequency with other TGD people (none to occasional, frequent to very frequent), education (below vocational diploma, vocational diploma or higher), employment (employed/self-employed, in training), and belonging to a minority other than gender or sexual minority. The analyses aimed to contribute to the discussion of a requirements profile for future e-health services tailored to the needs of TGD people.

### Procedures

The initial face-to-face interview with the study therapists at the study center comprised diagnostics and enrollment. Prospective participants completed baseline questionnaires at home *via* the e-health platform. Using a computer-generated code, participants were randomly allocated in a 1:1 ratio to the intervention or waitlist control group.

### Intervention

The core of the i^2^TransHealth intervention consisted of three elements: (1) 50-minute video consultations every two weeks, (2) an e-health platform, and (3) a network of outpatient physicians. During the intervention phase, service users were able to discuss TGD-specific as well as general mental health issues with their study therapists through video consultations, in the privacy of their own homes or another quiet, private space. The e-health platform in a responsive design, with public and protected areas, offered accessibility and featured informational texts, frequently asked questions, project publications, videos, tips, and links to TGD community sites. The protected area, exclusive to i^2^TransHealth service users with an account, facilitated confidential 1:1 chat conversations with their study therapists (with a 48-h response time on weekdays), document uploads, or a calendar. Questionnaires could be completed digitally at scheduled survey times (T0 and T1). The waitlist control group, during the initial four months, could only complete questionnaires but gained full access afterward. While i^2^TransHealth complements, rather than replaces, in-person TGD-informed healthcare, service users in crisis could contact our local network of trained outpatient physicians. Each of six sites in northern Germany had a primary care and psychiatric practice involved in i^2^TransHealth.

### Measures

Quantitative data for the secondary analysis were taken from the i^2^TransHealth intervention questionnaire battery (Nieder et al., 2022). In this article, only the measurements relevant to the research question are referred to. The primary outcome of the RCT is psychological distress according to the BSI-18, where we assumed a prevention of a worsening of psychological distress in the intervention group compared to the waitlist control group. The BSI-18 GSI, a total scale score, serves as the indicator, incorporating 18 items on somatization, depression, and anxiety. The 5-point Likert scale ranges from “not at all” to “very much” (*α* = 0.91 to 0.93 internal consistencies in German samples) (Franke et al., 2011, 2017). The BSI-18 GSI, ranging from 0 to 72 (with a higher GSI indicating more psychological distress), is instrumental in revealing the treatment response in alignment with the i^2^TransHealth concept.

### Statistical analysis

Subgroup analyses were performed for the primary outcome of the i^2^TransHealth study using the appropriate interaction test in order to capture the effect of the i^2^TransHealth intervention among specific subgroups of participants. The analyses were carried out in the intention-to-treat (ITT) population, encompassing all randomized participants. As part of sensitivity analyses, we performed subgroup analyses within the per-protocol (PP) population without major protocol violations, such as initiation of gender-affirming medical treatments outside the study elsewhere, study dropout, interim discontinuation and restart of the RCT condition during the study. All subgroup variables were measured at baseline and predefined in the statistical analysis plan of the i^2^TransHealth study, finalized before unmasking the study group allocation. Sociodemographic and intersectional variables investigated at baseline (T0), collected on a metric or multicategorical scale, were categorized for the subgroup analyses. Two or three subgroups per variable were built to achieve balanced distributions across the study population. Intersectional aspects were descriptively evaluated separately based on their frequency and relevance to the participants’ identity.

The subgroup analyses were performed using a linear regression model with the change from baseline at T1 in BSI-18 GSI as the dependent variable, the study group (intervention group vs. control group) as the independent variable and the baseline measurement of BSI-18 GSI as a covariate. To assess the intervention effect in the pre-specified subgroups, the interaction between the study group and the corresponding subgroup was included in the regression model, respectively.

In a further (post-hoc) sensitivity analysis, additional covariates were added to the primary analysis model that included the subgroup variables mentioned above.

A masked researcher at the Institute for Sex Research, Sexual Medicine and Forensic Psychiatry (J.R.) monitored, exported, and processed the T0 and T1 data, while independent researchers at the Institute of Medical Biometry and Epidemiology performed statistical analyses after unmasking. All applicable statistical tests were two-sided, and all analyses were carried out in an explorative manner without adjustment for multiple testing. SPSS 28 (IBM Corporation, NY, USA) and Stata 17 (StataCorp LLC, TX, USA) were used for data preparation and statistical analysis.

## Results

### Baseline characteristics

Descriptive statistics for the baseline measurement of the analysis population of all 168 participants with complete T1 follow-up, excluding dropouts, are shown in Table 1 (see Supplementary Tables S1 and S2 for an overview of the ITT and PP populations). Comparison of RCT groups shows they were well balanced on sociodemographic and intersectional variables. With regard to intersectional aspects, participants were able to indicate, if applicable, how important this aspect of their identity was to them, and the frequencies of these response options (“not important to me,” “only somewhat important to me,” or “very important”) are additionally listed.

**Table 1.** Baseline characteristics of participants by RCT group for all individuals in the analysis population, defined as those in the ITT population with a 4-month follow-up of the primary outcome.

		RCT group		
		Control (*N* = 80 of 84)	Intervention (*N* = 88 of 90)	Total (*N* = 168 of 174)
**Baseline characteristics**				
Age	Mean ± SD	27.3 ± 11.3	26.4 ± 8.6	26.8 ± 10.0
	Median [IQR]	23.0 [19.0; 29.5]	23.5 [20.0; 30.0]	23.0 [20.0; 30.0]
	Range	18.0–60.0	18.0–59.0	18.0–60.0
Sex assigned at birth (female)		43/80 (53.75%)	50/88 (56.82%)	93/168 (55.36%)
Gender identity	trans man/trans masculine	31/80 (38.75%)	42/88 (47.73%)	73/168 (43.45%)
	trans woman/trans feminine	27/80 (33.75%)	30/88 (34.09%)	57/168 (33.93%)
	non-binary	22/80 (27.50%)	16/88 (18.18%)	38/168 (22.62%)
Residence size	rural/small-town	37/80 (46.25%)	41/86 (47.67%)	78/166 (46.99%)
	non-rural	43/80 (53.75%)	45/86 (52.33%)	88/166 (53.01%)
	I cannot or do not want to answer	0/80 (0.00%)	2/88 (2.27%)	2/168 (1.19%)
Education	less than vocational diploma	43/80 (53.75%)	56/88 (63.64%)	99/168 (58.93%)
	vocational diploma/above	37/80 (46.25%)	32/88 (36.36%)	69/168 (41.07%)
Employment	employed or self-employed	35/71 (49.30%)	38/81 (46.91%)	73/150 (48.67%)
	in training (school, university, vocational training)	36/71 (50.70%)	41/81 (50.62%)	77/150 (51.33%)
	I cannot or do not want to answer	9/80 (11.25%)	9/88 (10.23%)	18/168 (10.71%)
Ethnicity (self-identification as Person of Color)	applicable	7/80 (8.75%)	8/88 (9.10%)	15/168 (8.93%)
	not important to me	3/80 (3.75%)	0/88 (0.00%)	3/168 (1.79%)
	only somewhat important to me	2/80 (2.50%)	4/88 (4.55%)	6/168 (3.57%)
	very important	2/80 (2.50%)	4/88 (4.55%)	6/168 (3.57%)
Religious minority	applicable	9/80 (11.25%)	4/88 (4.55%)	13/168 (7.74%)
	not important to me	2/80 (2.50%)	1/88 (1.14%)	3/168 (1.79%)
	only somewhat important to me	6/80 (7.50%)	2/88 (2.27%)	8/168 (4.76%)
	very important	1/80 (1.25%)	1/88 (1.14%)	2/168 (1.19%)
Sexual minority	applicable	70/80 (87.50%)	77/88 (87.50%)	147/168 (87.50%)
	not important to me	18/80 (22.50%)	23/88 (26.14%)	41/168 (24.40%)
	only somewhat important to me	24/80 (30.00%)	22/88 (25.00%)	46/168 (27.38%)
	very important	28/80 (35.00%)	32/88 (36.36%)	60/168 (35.71%)
Gender minority	applicable	55/80 (68.75%)	61/88 (69.32%)	116/168 (69.05%)
	not important to me	11/80 (13.75%)	15/88 (17.05%)	26/168 (15.48%)
	only somewhat important to me	14/80 (17.50%)	15/88 (17.01%)	29/168 (17.26%)
	very important	30/80 (37.50%)	31/88 (35.23%)	61/168 (36.31%)
Disability (self-reported)	applicable	16/80 (20.00%)	14/88 (15.91%)	30/168 (17.86%)
	not important to me	4/80 (5.00%)	4/88 (4.55%)	8/168 (4.76%)
	only somewhat important to me	8/80 (10.00%)	8/88 (9.10%)	16/168 (9.52%)
	very important	4/80 (5.00%)	2/88 (2.27%)	6/168 (3.57%)
Other non-specified minority (self-reported)	applicable	15/80 (18.75%)	13/88 (14.77%)	28/168 (16.67%)
	not important to me	6/80 (7.50%)	3/88 (3.41%)	9/168 (5.36%)
	only somewhat important to me	4/80 (5.00%)	3/88 (3.41%)	7/168 (4.17%)
	very important	5/80 (6.25%)	7/88 (7.95%)	12/168 (7.14%)
Do you have current or past contact with TGD people?	Not at all to occasionally	46/79 (58.23%)	59/87 (67.82%)	105/166 (63.25%)
	Frequent to very frequent	33/79 (41.77%)	28/87 (32.18%)	61/166 (36.75%)
	I cannot or do not want to answer	1/80 (1.25%)	1/88 (1.14%)	2/168 (1.19%)

Sex assigned at birth was either male or female. Gender identity was categorized and included a multiple response requestion. All participant regions, regardless of their residence size, are considered distant from specialized care for TGD people and where therefore included as they are structurally disconnected from a corresponding healthcare service. Vocational diploma corresponds to the German Fachabitur (a higher education qualification from upper secondary level).

**Table 2.** Primary outcome by RCT group (raw scores all timepoints, analysis population).

		RCT group		
		Control (*N* = 80 of 84)	Intervention (*N* = 88 of 90)	Total (*N* = 168 of 174)
**Primary outcome**				
BSI-18 Global Severity Index (GSI) (T0)	Mean ± SD	12.55 ± 10.80	13.09 ± 8.74	12.83 ± 9.75
	Median [IQR]	9.00 [5.00; 19.50]	11.00 [7.50; 17.00]	10.00 [6.00; 18.00]
	Range	0.00–53.00	0.00–44.00	0.00–53.00
BSI-18 Global Severity Index (GSI) (T1)	Mean ± SD	14.95 ± 11.84	12.39 ± 9.56	13.61 ± 10.75
	Median [IQR]	12.50 [6.00; 19.50]	10.50 [5.50; 15.00]	11.00 [6.00; 18.00]
	Range	0.00–50.00	0.00–52.00	0.00–52.00
	Missing	4/84 (4.76%)	2/90 (2.22%)	6/174 (3.45%)

### Primary outcome

Table 2 shows the raw scores of the BSI-18 GSI at all time points, demonstrating good internal consistency (*α* = 0.89 at T0, *α* = 0.91 at T1). At baseline (T0), the BSI-18 data were complete; at T1 follow-up, there were missing values (3.45%). As the rate of missing values was less than 5%, no values were imputed as prespecified in the statistical analysis plan. For the analyses of the primary outcome, *n* = 168 TGD participants were ultimately included in the analysis population. The main analyses of response to i^2^TransHealth treatment have been reported in detail elsewhere (Nieder et al., 2024).

### Subgroup analyses and adjusted primary analysis

In the following, we present the results of each subgroup analysis in term of the primary outcome (change from baseline at T1 in BSI-18 GSI) of the i^2^TransHealth study as well as the results of the adjusted primary analysis.

**Figure 2.**
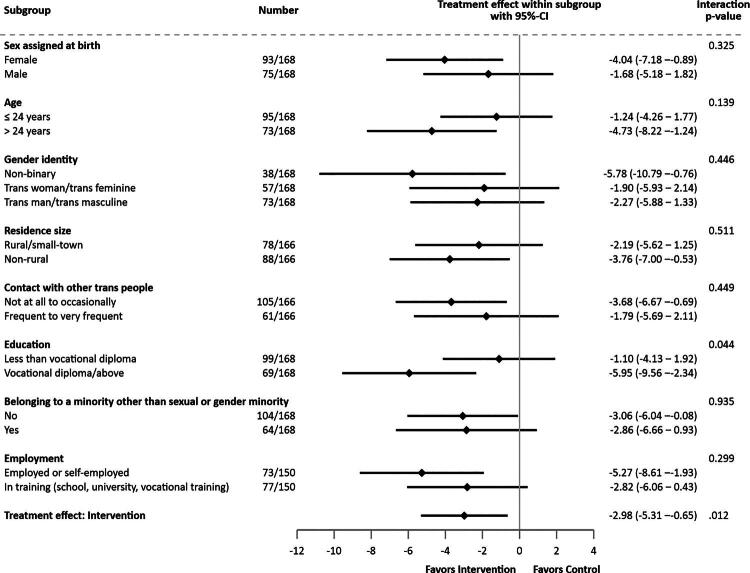
Forest plot of the subgroup analyses (analysis population).

As a results of the subgroup analysis, the baseline adjusted treatment effect within each subgroup and its corresponding 95%-CI as well as the interaction *p*-value between the study group and the specific subgroup are reported (Figure 2 and Supplementary Table S3). For the sake of simplicity, results for the PP population and the adjusted primary analysis are presented in the Supplement (Supplementary Table S4, Figure S1 and S2).

The analysis population included *n* = 168 TGD participants, with subgroup analyses of *n* = 166 TGD participants each for the variable of residence size and contact with other TGD people, and a subgroup analysis of *n* = 150 TGD participants for the variable employment due to missing values on the respective baseline variable. The subgroup analysis of the primary outcome revealed a clinically relevant group difference in education level, with a treatment effect of −1.10 [−4.13; 1.92] for participants with less than a vocational diploma and −5.95 [−9.56; −2.34] for those above vocational diploma (adjusted treatment effect difference: −4.84 [−9.56; −0.13], *p*_interaction_ = 0.044).

Individuals aged 25 and above experienced a greater benefit with a treatment effect of −4.73 [−8.22; −1.24] compared to those under 25 with a treatment effect of −1.24 [−4.26; −1.77] (adjusted difference of treatment effect −3.49 [−8.11; 1.14]; *p*_interaction_ = .139). Trans masculine and trans feminine individuals benefited less than non-binary individuals (adjusted difference of treatment effect 3.50 [−2.70; 9.70] respectively 3.88 [−2.58; 10.33]; *p*_interaction_ = .446).

Between the two employment subgroups, the treatment effect differed by 2.46 ([−2.20; 7.11]; *p*_interaction_ = .299) points. Similarly, no relevant specific subgroup differences were noted for sex assigned at birth (adjusted difference of treatment effect 2.36 [−2.35; −7.07]; *p*_interaction_ = .325), residence size (adjusted difference of treatment effect −1.58 [−6.30; 3.14]; *p*_interaction_ = .511), contact with other TGD people (adjusted difference of treatment effect 1.89 [−3.03; 6.81]; *p*_interaction_ = .449), and belonging to a minority other than sexual or gender minority (adjusted difference of treatment effect 0.20 [−4.62; 5.02]; *p*_interaction_ = .935).

Using the PP population of *n* = 161 TGD participants, the subgroup analyses were repeated with the number of cases reduced to *n* = 159 for the variable residence size, *n* = 160 for the variable contact with other TGD people and *n* = 145 for the variable employment. These sensitivity analyses showed that the results were robust across both population definitions. Overall, the subgroups related to age, education, and employment were similarly distributed over the two RCT groups.

The result of the adjusted primary analysis showed that the addition of further covariates slightly increased the treatment effect between the study groups compared to the primary analysis of the i^2^TransHealth study (treatment effect (95% CI): −3.66 [−6.03; −1.29]; *p* = .003) (Supplementary Figure S2).

## Discussion

### Summary of findings

The main objective of this article is to present the findings of the explorative subgroup analyses of the primary outcome of the RCT on the e-health intervention i^2^TransHealth for adult TGD people. Our analyses of the effectiveness of the e-health intervention are among the first intervention studies of their kind in TGD-informed healthcare. In addition, we are the first to examine how different subgroup characteristics relate to the prevention of worsening in psychological distress, which is a marker of treatment success of e-health for TGD people. The added value of this study is to be able to make statements about not only whether e-health is effective for TGD people, but for whom exactly it may be particularly effective.

We considered assigned sex at birth, age, gender identity, residence size, contact with other TGD people, education, employment, and belonging to a marginalized group other than sexual and gender minority. Our findings, in addition to the overall effectiveness of i^2^TransHealth, suggested that education was important in the responsiveness to e-health treatment services. Service users with higher levels of education had greater reductions in psychological distress than those with lower levels of education (see Figure 2). Higher education often correlates with advantageous living conditions (e.g., better prospects for health; Crissman et al., 2017), potentially facilitating the successful application of health-promoting measures such as e-health services. Notably, TGD people in educational settings often face additional challenges arising from transphobic bullying and discrimination, potentially exacerbating psychological distress and impacting academic performance (De Pedro et al., 2018; Eisenberg et al., 2019; Johns et al., 2019; Kosciw et al., 2009, 2015; Parodi et al., 2022; Wang et al., 2020). Regarding age, the results show that age is likely a treatment-relevant factor and that belonging to a higher age group (i.e., older than 24 years) could be health-promoting in our treatment setting. In addition to education and age, the subgroup analyses for employment as another relevant sociodemographic factor suggest that each variable contributes slightly differently to the response to the e-health intervention. Rather than a single factor, it appears to be a combination of various overall life situations of TGD people (whether temporary or permanent) that influences the outcomes. Our exploratory analyses suggest that various sociodemographic factors could be important in shaping the response to the intervention in different ways.

No clinically relevant group differences emerged for assigned sex at birth, different sizes of residence, contact with other TGD people, and belonging to a marginalized group besides the TGD community (see Figure 2 and Supplementary Table 3). For assigned sex at birth and gender identity, this was to be expected, as our e-health intervention was open to all TGD people and no gender-specific advantages or disadvantages for e-health can be derived from the literature (Radix et al., 2022; Renner et al., 2022; Smalley et al., 2022; Stoehr et al., 2022; Wong et al., 2022). The non-binary service user data, on the other hand, showed high variability in psychological distress, but this could just be a chance result. The results on connection to the TGD community in terms of contact with other TGD people were surprising. It would be expected that especially isolated TGD service users, who have no or little community contact, would benefit more clearly by being connected to a TGD-specific e-health service (James et al., 2017; McCann & Sharek, 2016; Renner et al., 2021; Rosenkrantz et al., 2017). It should be emphasized, however, that due to the COVID-19 pandemic, social contacts were limited for all TGD people and shifted more to the digital (Kidd et al., 2021; Koehler et al., 2023). Another possible interpretation of this finding could be that a lack or low level of contact with other TGD people alone is not automatically a proxy for isolation, as TGD people can also have a functioning social network without desiring or seeking contact with the TGD community (Eyssel et al., 2017; Valentine & Shipherd, 2018). Independently of this subgroup evaluation, however, in an upcoming publication of our qualitative process evaluation of i^2^TransHealth, we observed concentrated strong anti-TGD resentment in remote areas (Schmidt et al., n.d.). The importance of an intersectional aspect to an individual’s identity varied widely (see Table 1). However, with respect to marginalized groups other than sexual or gender minorities (i.e., ethnic minority, religious minority, disability, other non-specified minority), the subgroup analyses indicated that potential intersectional discrimination does not necessarily alter a treatment effect, particularly within an already marginalized group like TGD people.

### Implications and future directions

Based on the results of the study, the Innovation Committee at the Federal Joint Committee (G-BA), which funded the study but was not involved in the study design, data collection, analysis, interpretation, or report writing, has recommended the integration of i^2^TransHealth into standard care. For this reason, the G-BA has forwarded the project results to national health insurance funds and relevant professional associations in Germany (detailed reports are available in German at https://innovationsfonds.g-ba.de/beschluesse/i2transhealth.253). Our results of the present subgroup analyses provide valuable insights and potential directions for further research and development of e-health services for TGD people. Our findings suggest that e-health may be a helpful and effective treatment modality for TGD people, with education levels influencing the choice and responsiveness to such services, aligning with comparable studies (Phanuphak et al., 2020).

Health promotion initiatives aimed at TGD people are recommended to consider diverse age and educational backgrounds. Addressing this variability could have a stabilizing effect for individuals and increase equity for treatment success and positive health outcomes. Leveraging the adaptability of e-health through blended care, tailored to the individual needs of a TGD person, could be a practical approach (Erbe et al., 2017; Wentzel et al., 2016). Reaching hard-to-reach populations in healthcare in general is a challenge. In Germany, a vocational diploma or higher level of education offers many opportunities for societal and professional participation, making it relatively easier for people with a high level of education to seek out and benefit from healthcare services. People with a lower level of education, on the other hand, may face difficulties in various areas of life that are not directly apparent from their level of education (Burgwal et al., 2019; European Union Agency for Fundamental Rights, 2020; Pöge et al., 2020). In this respect, our subgroup analyses can be seen as another observation of the inverse caw law (i.e., inequalities in care are often to the disadvantage of people with a lower socioeconomic status) (Cookson et al., 2021). An intersectional perspective in healthcare research proves to be indispensable (Bauer, 2014; Gkiouleka et al., 2018; Wemrell et al., 2021). Overall, our findings underscore the importance of identifying and addressing preexisting social inequalities within TGD communities in the best possible way in e-health approaches. Strategies to enhance an e-health treatment outcome for all TGD service groups could include attention to diverse outreach in different channels, awareness of different target groups and their interests, tailored treatment style with online, offline or a mix of these options (Renner et al., 2022). Engaging service users in discussions about further support services during treatment can help refine care delivery and optimize the use of e-health elements.

Concerning the current lack of research and evidence-based e-health concepts in TGD-informed healthcare, we anticipate a growing demand for such services in the course of digitalization. Given this future perspective, longitudinal studies, including TGD people at various stages of transition, are crucial to understanding the needs of TGD communities. Future research should systematically collect socioeconomic variables and other important life-relevant conditions (Bauer, 2014; Bauer & Scheim, 2019; Gkiouleka et al., 2018; Wemrell et al., 2021) to develop a more comprehensive understanding of the impact of e-health services. One aim of e-health services is to alleviate challenges stemming from vulnerable socioeconomic conditions (e.g., costs due to lost working hours or long travel times), ultimately enhancing the precision and effectiveness of e-health interventions tailored to the individual life situations of TGD people.

### Strengths and limitations

This study marks the pioneering effort in conducting an RCT on e-health and TGD-informed healthcare in mental health. i^2^TransHealth holds a trailblazing status not only in the German-speaking world, but provides insights applicable to countries globally where e-health services have been implemented or are of interest. Distinguishing itself from mere observational studies assessing the acceptance of e-health modalities (Hertling et al., 2021; Russell et al., 2022; Sequeira et al., 2021; Silva et al., 2021), i^2^TransHealth is unique in its design as a TGD-specific e-health intervention implemented in the real world, allowing for a comprehensive assessment of its effectiveness. Different subgroups were identified and compared in terms of their response to an e-health treatment.

It should be noted that this study presented exploratory analyses, limiting the ability to draw causal conclusions. The RCT on i^2^TransHealth tested the equality of the two RCT groups with respect to the primary outcome psychological distress. Socioeconomic analyses were not possible in depth for these additional analyses at this point, but instead we refer to our health economic evaluations (Grochtdreis, König, Konnopka, et al., n.d.; Grochtdreis, König, Renner, et al., n.d.). The RCT, by design, focused on TGD people in an early phase of transition, precluding conclusions for those who seek follow-up appointments *via* e-health during the transition. While this approach was essential for evaluating an e-health service as the first point of access to TGD-informed healthcare, we encourage researchers and HCPs in future e-health endeavors to evaluate the suitability of e-health services for various transition phases.

Predefined subgroup variables guided the analyses, yet detailed comparisons were constrained by the aim to maintain subgroup balance. The wider age range in the 25-and-over group, influenced by a higher representation of young participants, hindered differentiation into distinct age categories. Intersectionality was addressed through a global minority category and the analysis may have oversimplified the nuanced impact of additive minority stress (Hendricks & Testa, 2012; Lefevor et al., 2019; Valentine & Shipherd, 2018; Vincent, 2018). The strength of the study lies in its inclusion of TGD people from remote areas often neglected in research (Renner et al., 2021), highlighting the heterogeneity within the TGD community. These considerations underscore the need for HCPs to empower their TGD service users in a diversity-sensitive manner, acknowledging the varied experiences within this population (de Vries et al., 2020; McCann & Sharek, 2016).

## Conclusion

This study aimed to conduct a secondary analysis of the e-health intervention i^2^TransHealth for TGD people, identifying potential adaptations and guiding further research based on subgroup-specific effects. Our findings suggest that i^2^TransHealth is effective for TGD people, but that TGD service users with higher levels of education tend to derive greater benefits. To ensure that e-health services are inclusive and do not unintentionally reinforce inequalities, it would be useful to explore strategies to better reach and support TGD people with lower levels of education. This could include developing e-health interventions that are user-friendly, culturally competent, and accessible to individuals from different educational backgrounds, emphasizing that education level is not considered as a flaw or barrier. Rather, it can be listed as one of the aspects that is a helpful consideration when developing and adapting e-health services to ensure accessibility for all. These findings contribute to our understanding of the effectiveness of e-health in TGD-informed healthcare, providing a basis for advancing treatment services. E-health has the potential to reach TGD people from remote areas, addressing longstanding accessibility challenges. Remaining questions center on the expansion of e-health services beyond mental health to encompass interdisciplinary TGD-informed healthcare disciplines (Renner et al., 2022; Smalley et al., 2022). Rigorous research with RCTs is essential for evidence-based e-health care, particularly in bridging existing gaps in care. Broader research is warranted to determine which treatment steps of a transition necessitate in-person interactions and which can be optionally conducted online, with on-site support available in emergencies. Large-scale, longer longitudinal studies are essential to understand personalized e-health preferences in diverse TGD subgroups, informing the development of inclusive interventions tailored to their needs.

## Supplementary Material

Supplemental Material

## Data Availability

The study protocol has been previously published. All proposals for data use need approval by the study team before any data are released. Rights to use the raw data will remain with the study team. De-identified data, analysis syntax, and the statistical analysis plan will be made available on reasonable request from the last author (tnieder@uke.de), and informed consent forms are available in German only. Data collected for the study will be made available in de-identified form upon request. However, code or analysis syntax will not be shared. Data access requests, which can be submitted by email from the date of publication, must include a detailed description of the research question and aims. Data sharing will be authorized upon approval of the request and execution of a signed data access agreement for scientific use only.
