# Supplementary material for: Which transgender and gender diverse groups benefit most from E-health? Subgroup analyses of the randomized controlled trial i2TransHealth in Germany
Source: Int J Transgend Health. 2025 Feb 22;27(2):824–39. doi: 10.1080/15532739.2025.2465710 (PMC13015102; doi:10.1080/15532739.2025.2465710)

**Supplement**

Which Transgender and Gender Diverse Groups Benefit Most from E-Health? Subgroup Analyses of the Randomized Controlled Trial i²TransHealth in Germany

**Supplementary Table 1. Baseline characteristics of participants by RCT group (ITT population).**

|  | | **RCT group** | |  |
| --- | --- | --- | --- | --- |
|  |  | **Control (N=84)** | **Intervention (N=90)** | **Total (N=174)** |
| **Baseline characteristics** | |  |  |  |
| Age | Mean ± SD | 27.1 ± 11.1 | 27.0 ± 9.4 | 27.0 ± 10.2 |
|  | Median [IQR] | 23.0 [19.0;29.5] | 24.0 [20.0;30.0] | 23.5 [20.0;30.0] |
|  | Range | 18.0-60.0 | 18.0-59.0 | 18.0-60.0 |
| Sex assigned at birth (female) | | 46/84 (54.76%) | 50/90 (55.56%) | 96/90 (55.17%) |
| Gender identity | trans man/trans masculine | 34/84 (40.48%) | 42/90 (46.67%) | 76/90 (43.68%) |
|  | trans woman/trans feminine | 27/84 (32.14%) | 32/90 (35.56%) | 59/90 (33.91%) |
|  | non-binary | 23/84 (27.38%) | 16/90 (17.78%) | 39/90 (22.41%) |
| Residence size | rural/small-town | 39/83 (46.99%) | 42/88 (47.73%) | 81/171 (47.39%) |
|  | non-rural | 44/83 (53.01%) | 46/88 (52.27%) | 90/171 (52.63%) |
|  | I cannot or do not want to answer | 1/84 (1.19%) | 2/90 (2.22%) | 3/174 (1.72%) |
| Education | less than vocational diploma | 46/84 (54.76%) | 56/90 (62.22%) | 102/174 (58.62%) |
|  | vocational diploma/above | 38/84 (45.24%) | 34/90 (37.78%) | 72/174 (41.38%) |
| Employment | employed or self-employed | 36/73 (49.32%) | 40/81 (49.38%) | 76/154 (49.35%) |
|  | in training (school, university, vocational training) | 37/73 (50.68%) | 41/81 (50.62%) | 78/154 (50.65%) |
|  | I cannot or do not want to answer | 11/84 (13.10%) | 9/90 (10.00%) | 20/174 (11.49%) |
| Ethnicity (self-identification as Person of Color) | applicable | 9/84 (10.71%) | 9/90 (10.00%) | 18/174 (10.34%) |
|  | not important to me | 4/84 (4.76%) | 1/90 (1.11%) | 5/174 (2.87%) |
|  | only somewhat important to me | 3/84 (3.57%) | 4/90 (4.44%) | 7/174 (4.02%) |
|  | very important | 2/84 (2.38%) | 4/90 (4.44%) | 6/174 (3.45%) |
| Religious minority | applicable | 10/84 (11.90%) | 4/90 (4.44%) | 14/174 (8.05%) |
|  | not important to me | 3/84 (3.57%) | 1/90 (1.11%) | 4/174 (2.30%) |
|  | only somewhat important to me | 6/84 (7.14%) | 2/90 (2.22%) | 8/174 (4.60%) |
|  | very important | 1/84 (1.19%) | 1/90 (1.11%) | 2/174 (1.15%) |
| Sexual minority | applicable | 74/84 (88.10%) | 78/90 (86.67%) | 152/174 (87.36%) |
|  | not important to me | 21/84 (25.00%) | 23/90 (25.56%) | 44/174 (25.29%) |
|  | only somewhat important to me | 25/84 (29.76%) | 22/90 (24.44%) | 47/174 (27.01%) |
|  | very important | 28/84 (33.33%) | 33/90 (36.67%) | 61/174 (35.06%) |
| Gender minority | applicable | 58/84 (69.05%) | 63/90 (70.00%) | 121/174 (69.54%) |
|  | not important to me | 11/84 (13.10%) | 16/90 (17.78%) | 27/174 (15.52%) |
|  | only somewhat important to me | 17/84 (20.24%) | 15/90 (16.67%) | 32/174 (18.39%) |
|  | very important | 30/84 (35.71%) | 32/90 (35.56%) | 62/174 (35.63%) |
| Disability (self-reported) | applicable | 16/84 (19.05%) | 14/90 (15.56%) | 30/174 (17.24%) |
|  | not important to me | 4/84 (4.76%) | 4/90 (4.44%) | 8/174 (4.60%) |
|  | only somewhat important to me | 8/84 (9.52%) | 8/90 (8.89%) | 16/174 (9.20%) |
|  | very important | 4/84 (4.76%) | 2/90 (2.22%) | 6/174 (3.45%) |
| Other non-specified minority (self-reported) | applicable | 16/84 (19.05%) | 13/90 (14.44%) | 29/174 (16.67%) |
|  | not important to me | 7/84 (8.33%) | 3/90 (3.33%) | 10/174 (5.75%) |
|  | only somewhat important to me | 4/84 (4.76%) | 3/90 (3.33%) | 7/174 (4.02%) |
|  | very important | 5/84 (5.95%) | 7/90 (7.78%) | 12/174 (6.90%) |
| Do you have current or past contact with TGD people? | Not at all to occasionally | 49/83 (59.04%) | 60/89 (67.42%) | 109/172 (63.37%) |
|  | Frequent to very frequent | 34/83 (40.96%) | 29/89 (32.58%) | 63/172 (36.63%) |
|  | I cannot or do not want to answer | 1/84 (1.19%) | 1/90 (1.11%) | 2/174 (1.15%) |
| BSI-18 Global Severity Index (GSI) (T0) | Mean ± SD | 12.52 ± 10.58 | 12.86 ± 8.78 | 12.70 ± 9.66 |
|  | Median [IQR] | 9.00 [5.00;18.50] | 11.00 [7.00;17.00] | 10.00 [6.00;17.00] |
|  | Range | 0.00-53.00 | 0.00-44.00 | 0.00-53.00 |

Notes. Vocational diploma corresponds to the German Fachabitur (a higher education qualification from upper secondary level).

**Supplementary Table 2. Baseline characteristics of participants by RCT group (PP population).**

|  | | **RCT group** | |  |
| --- | --- | --- | --- | --- |
|  |  | **Control (N=76)** | **Intervention (N=85)** | **Total (N=161)** |
| **Baseline characteristics** | |  |  |  |
| Age | Mean ± SD | 26.8 ± 11.1 | 26.3 ± 8.7 | 26.5 ± 9.9 |
|  | Median [IQR] | 22.5 [19.0;28.5] | 23.0 [20.0;30.0] | 23.0 [20.0;29.0] |
|  | Range | 18.0-60.0 | 18.0-59.0 | 18.0-60.0 |
| Sex assigned at birth (female) | | 42/76 (55.26%) | 50/85 (58.82%) | 92/161 (57.14%) |
| Gender identity | trans man/trans masculine | 31/76 (40.79%) | 42/85 (49.41%) | 73/161 (45.34%) |
|  | trans woman/trans feminine | 25/76 (32.89%) | 27/85 (31.76%) | 52/161 (32.30%) |
|  | non-binary | 20/76 (26.32%) | 16/85 (18.82%) | 36/161 (22.36%) |
| Residence size | rural/small-town | 35/76 (46.05%) | 39/83 (46.99%) | 74/159 (46.54%) |
|  | non-rural | 41/76 (53.95%) | 44/83 (53.01%) | 85/159 (53.46%) |
|  | I cannot or do not want to answer | 0/76 (0.00%) | 2/85 (2.35%) | 2/161 (1.24%) |
| Education | less than vocational diploma | 42/76 (55.26%) | 53/85 (62.35%) | 95/161 (59.01%) |
|  | vocational diploma/above | 34/76 (44.74%) | 32/85 (37.65%) | 66/161 (40.99%) |
| Employment | employed or self-employed | 32/67 (47.76%) | 37/78 (47.44%) | 69/145 (47.59%) |
|  | in training (school, university, vocational training) | 35/67 (52.24%) | 41/78 (52.56%) | 76/145 (52.41%) |
|  | I cannot or do not want to answer | 9/76 (11.84%) | 7/85 (8.24%) | 16/161 (9.94%) |
| Ethnicity (self-identification as Person of Color) | applicable | 6/76 (7.89%) | 8/85 (9.41%) | 14/161 (8.70%) |
|  | not important to me | 3/76 (3.95%) | 0/85 (0.00%) | 3/161 (1.86%) |
|  | only somewhat important to me | 2/76 (2.63%) | 4/85 (4.71%) | 6/161 (3.73%) |
|  | very important | 1/76 (1.32%) | 4/85 (4.71%) | 5/161 (3.11%) |
| Religious minority | applicable | 9/76 (11.84%) | 4/85 (4.71%) | 13/161 (8.07%) |
|  | not important to me | 2/76 (2.63%) | 1/85 (1.18%) | 3/161 (1.86%) |
|  | only somewhat important to me | 6/76 (7.89%) | 2/85 (2.35%) | 8/161 (4.97%) |
|  | very important | 1/76 (1.32%) | 1/85 (1.18%) | 2/161 (1.24%) |
| Sexual minority | applicable | 66/76 (86.84%) | 74/85 (87.06%) | 140/161 (86.96%) |
|  | not important to me | 18/76 (23.68%) | 22/85 (25.88%) | 40/161 (24.84%) |
|  | only somewhat important to me | 21/76 (27.63%) | 21/85 (24.71%) | 42/161 (26.09%) |
|  | very important | 27/76 (35.53%) | 31/85 (36.47%) | 58/161 (36.02%) |
| Gender minority | applicable | 51/76 (67.11%) | 59/85 (69.41%) | 110/161 (68.32%) |
|  | not important to me | 9/76 (11.84%) | 15/85 (17.65%) | 24/161 (14.91%) |
|  | only somewhat important to me | 13/76 (17.11%) | 14/85 (16.47%) | 27/161 (16.77%) |
|  | very important | 29/76 (38.16%) | 30/85 (35.29%) | 59/161 (36.65%) |
| Disability (self-reported) | applicable | 14/76 (18.42%) | 14/85 (16.47%) | 28/161 (17.39%) |
|  | not important to me | 4/76 (5.26%) | 4/85 (4.71%) | 8/161 (4.97%) |
|  | only somewhat important to me | 7/76 (9.21%) | 8/85 (9.41%) | 15/161 (9.32%) |
|  | very important | 4/80 (5.00%) | 2/88 (2.27%) | 6/168 (3.57%) |
| Other non-specified minority (self-reported) | applicable | 13/76 (17.11%) | 12/85 (14.12%) | 25/161 (15.53%) |
|  | not important to me | 5/76 (6.58%) | 3/85 (3.53%) | 8/161 (4.97%) |
|  | only somewhat important to me | 4/76 (5.26%) | 3/85 (3.53%) | 7/161 (4.35%) |
|  | very important | 4/76 (5.26%) | 6/85 (7.06%) | 10/161 (6.21%) |
| Do you have current or past contact with TGD people? | Not at all to occasionally | 42/75 (56.00%) | 57/85 (67.06%) | 99/160 (61.88%) |
|  | Frequent to very frequent | 33/75 (44.00%) | 28/85 (32.94%) | 61/160 (38.13%) |
|  | I cannot or do not want to answer | 1/76 (1.32%) | 0/85 (0.00%) | 1/161 (0.62%) |

Notes. Vocational diploma corresponds to the German Fachabitur (a higher education qualification from upper secondary level).

**Supplementary Table 3. Subgroup analyses for the primary outcome (BSI-18 GSI) in the analysis population, defined as those in the ITT population with a complete 4-month follow up.**

|  | **n/N (%)** | **Control** | **Intervention** | **Treatment effect within subgroup** | **Difference of treatment effect between subgroup** | **p-value** |
| --- | --- | --- | --- | --- | --- | --- |
| **Sex assigned at birth** | | | | | | |
| female | 93/168 (55%) | 2.53 | -1.32 | -4.04 [-7.18;-0.89] | ref | 0.325 |
| male | 75/168 (45%) | 2.24 | .11 | -1.68 [-5.18;1.82] | 2.36 [-2.35; 7.07] |  |
| **Age categorized** | | | | | | |
| age 18 to 24 | 95/168 (57%) | 2.63 | 1.77 | -1.24 [-4.26;1.77] | ref | 0.139 |
| age 25 to 60 | 73/168 (43%) | 2.06 | -3.54 | -4.73 [-8.22;-1.24] | -3.49 [-8.11; 1.14] |  |
| **Gender identity** | | | | | | |
| trans man/trans masculine | 73/168 (43%) | 2.00 | 2.00 | -2.27 [-5.88;1.33] | 3.50 [-2.70;9.70] | 0.446 |
| trans woman/trans feminine | 57/168 (34%) | 2.85 | .40 | -1.90 [-5.93;2.14] | 3.88 [-2.58;10.34] |  |
| non-binary | 38/168 (23%) | 2.41 | -2.38 | -5.78 [-10.79;-0.76] | ref |  |
| **Residence size** | | | | | | |
| rural/small-town | 78/166 (47%) | 3.03 | .95 | -2.19 [-5.62;1.25] | ref | 0.511 |
| non-rural | 88/166 (53%) | 1.86 | -2.29 | -3.76 [-7.00;-0.53] | -1.58 [-6.30; 3.14] |  |
| **Contact with other TGD people** | | | | | | |
| not at all to occasionally | 105/166 (63%) | 2.74 | -1.20 | -3.68 [-6.67;-0.69] | ref | 0.449 |
| frequent to very frequent | 61/166 (37%) | 1.91 | .21 | -1.79 [-5.69;2.11] | 1.89 [-3.03; 6.81] |  |
| **Education** | | | | | | |
| less than vocational diploma | 99/168 (59%) | 1.65 | .64 | -1.10 [-4.13;1.92] | ref | 0.044 |
| vocational diploma/above | 69/168 (41%) | 3.27 | -3.06 | -5.95 [-9.56;-2.34] | -4.84 [-9.56; -0.13] |  |
| **Employment** | | | | | | |
| employed or self-employed | 73/150 (49%) | 2.74 | -3.03 | -5.27 [-8.61;-1.93] | ref | 0.299 |
| in training (school, university, vocational training) | 77/150 (51%) | 2.72 | -.15 | -2.82 [-6.06;0.43] | 2.46 [-2.20; 7.11] |  |
| **Belonging to a minority other than sexual or gender minority** | | | | | | |
| no | 104/168 (62%) | 2.88 | -.29 | -3.06 [-6.04;-0.08] | ref | 0.935 |
| yes | 64/168 (38%) | 1.65 | -1.39 | -2.86 [-6.66;0.93] | 0.20 [-4.62; 5.02] |  |
| **All participants** | 168/174 (97%) | 2.40 | -.70 | -2.98 [-5.31;-0.65] |  | 0.012 |

*Note*. The employment variable was included in subgroup analyses after unmasking.

**Supplementary Table 4. Subgroup analyses for the primary outcome (BSI-18 GSI) in the PP population.**

|  | **n/N (%)** | **Control** | **Intervention** | **Treatment effect within subgroup** | **Difference of treatment effect between subgroup** | **p-value** |
| --- | --- | --- | --- | --- | --- | --- |
| **Sex assigned at birth** | | | | | | |
| female | 92/161 (57%) | 2.64 | -1.32 | -4.10 [-7.30;-0.90] | ref | 0.464 |
| male | 69/161 (43%) | 2.71 | -.06 | -2.28 [-5.98;1.41] | 1.82 [-3.07; 6.71] |  |
| **Age categorized** | | | | | | |
| age 18 to 24 | 93/161 (58%) | 2.72 | 1.74 | -1.34 [-4.42;1.74] | ref | 0.092 |
| age 25 to 60 | 68/161 (42%) | 2.59 | -3.79 | -5.47 [-9.13;-1.80] | -4.13 [-8.93; 0.68] |  |
| **Gender identity** | | | | | | |
| trans man/trans masculine | 73/161 (45%) | 2.00 | 2.00 | -2.30 [-5.94;1.35] | 3.64 [-2.72;10.00] | 0.512 |
| trans woman/trans feminine | 52/161 (33%) | 3.64 | .22 | -2.79 [-7.06;1.48] | 3.15 [-3.60;9.89] |  |
| non-binary | 36/161 (22%) | 2.50 | -2.38 | -5.94 [-11.11;-0.76] | ref |  |
| **Residence size** | | | | | | |
| rural/small-town | 74/159 (47%) | 3.60 | .90 | -2.81 [-6.37;0.75] | ref | 0.675 |
| non-rural | 85/159 (53%) | 1.88 | -2.39 | -3.85 [-7.18;-0.52] | -1.04 [-5.91; 3.84] |  |
| **Contact with other TGD people** | | | | | | |
| not at all to occasionally | 99/160 (62%) | 3.26 | -1.30 | -4.22 [-7.34;-1.10] | ref | 0.339 |
| frequent to very frequent | 61/160 (38%) | 1.91 | .21 | -1.78 [-5.72;2.15] | 2.44 [-2.58; 7.46] |  |
| **Education** | | | | | | |
| less than vocational diploma | 95/161 (59%) | 2.00 | .57 | -1.44 [-4.55;1.68] | ref | 0.053 |
| vocational diploma/above | 66/161 (41%) | 3.50 | -3.06 | -6.23 [-9.95;-2.51] | -4.79 [-9.65; 0.06] |  |
| **Employment** | | | | | | |
| employed or self-employed | 69/145 (48%) | 3.28 | -3.19 | -6.02 [-9.46;-2.58] | ref | 0.195 |
| in training (school, university, vocational training) | 76/145 (52%) | 2.86 | -.15 | -2.90 [-6.17;0.37] | 3.12 [-1.62; 7.87] |  |
| **Belonging to a minority other than sexual or gender minority** | | | | | | |
| no | 100/161 (62%) | 2.94 | -.40 | -3.21 [-6.28;-0.14] | ref | 0.904 |
| yes | 61/161 (38%) | 2.24 | -1.47 | -3.51 [-7.44;0.42] | -0.30 [-5.29; 4.68] |  |
| **All participants** | 161/174 (93%) | 2.67 | -.80 | -3.32 [-5.73;-0.92] |  | 0.007 |

*Note*. The employment variable was included in subgroup analyses after unmasking.

**Supplementary Fig. 1. Forest plot of the subgroup analyses (PP population).**


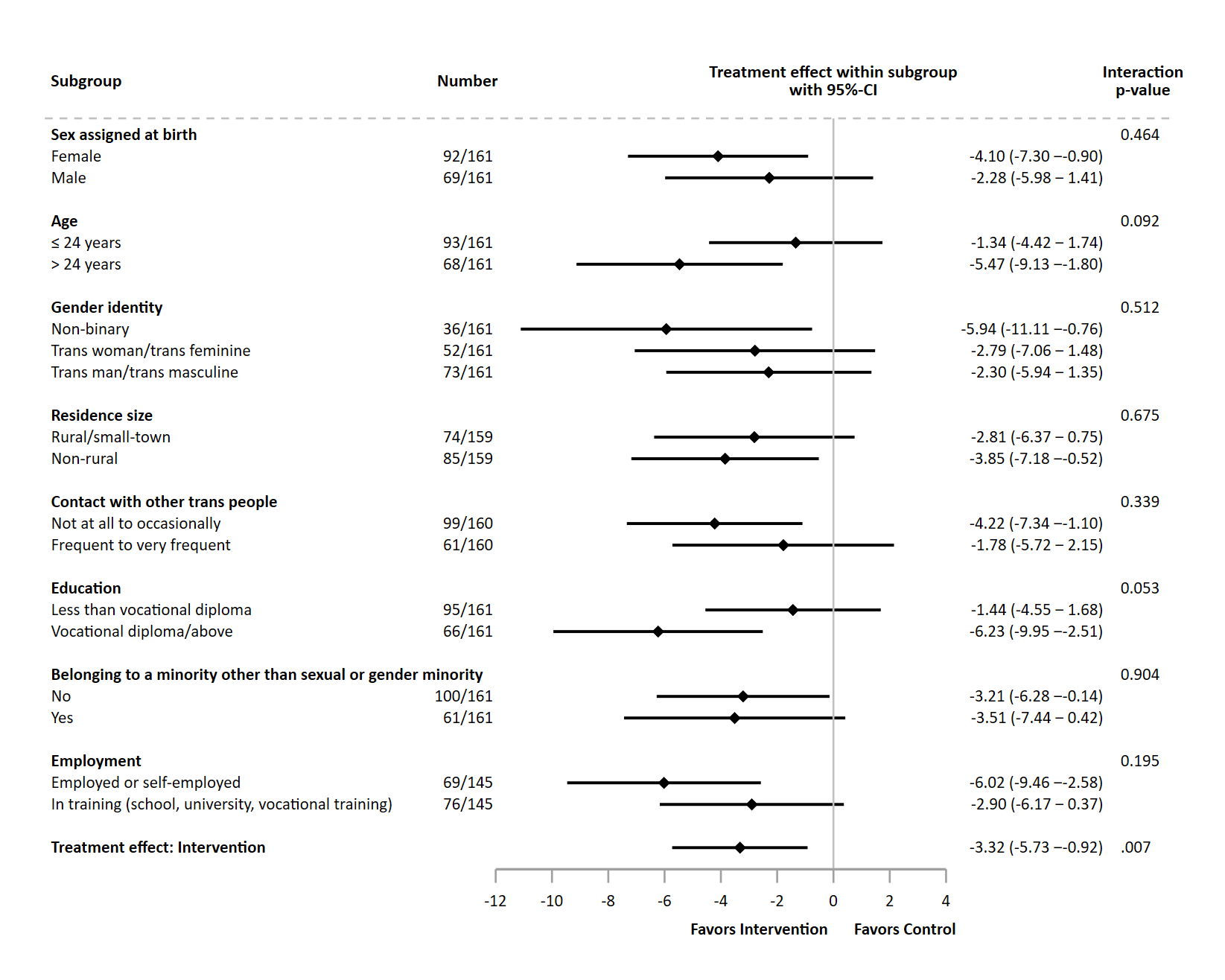


**Supplementary Fig. 2. Forest plot of the adjusted primary analysis (full model output, N=147, ITT population).**


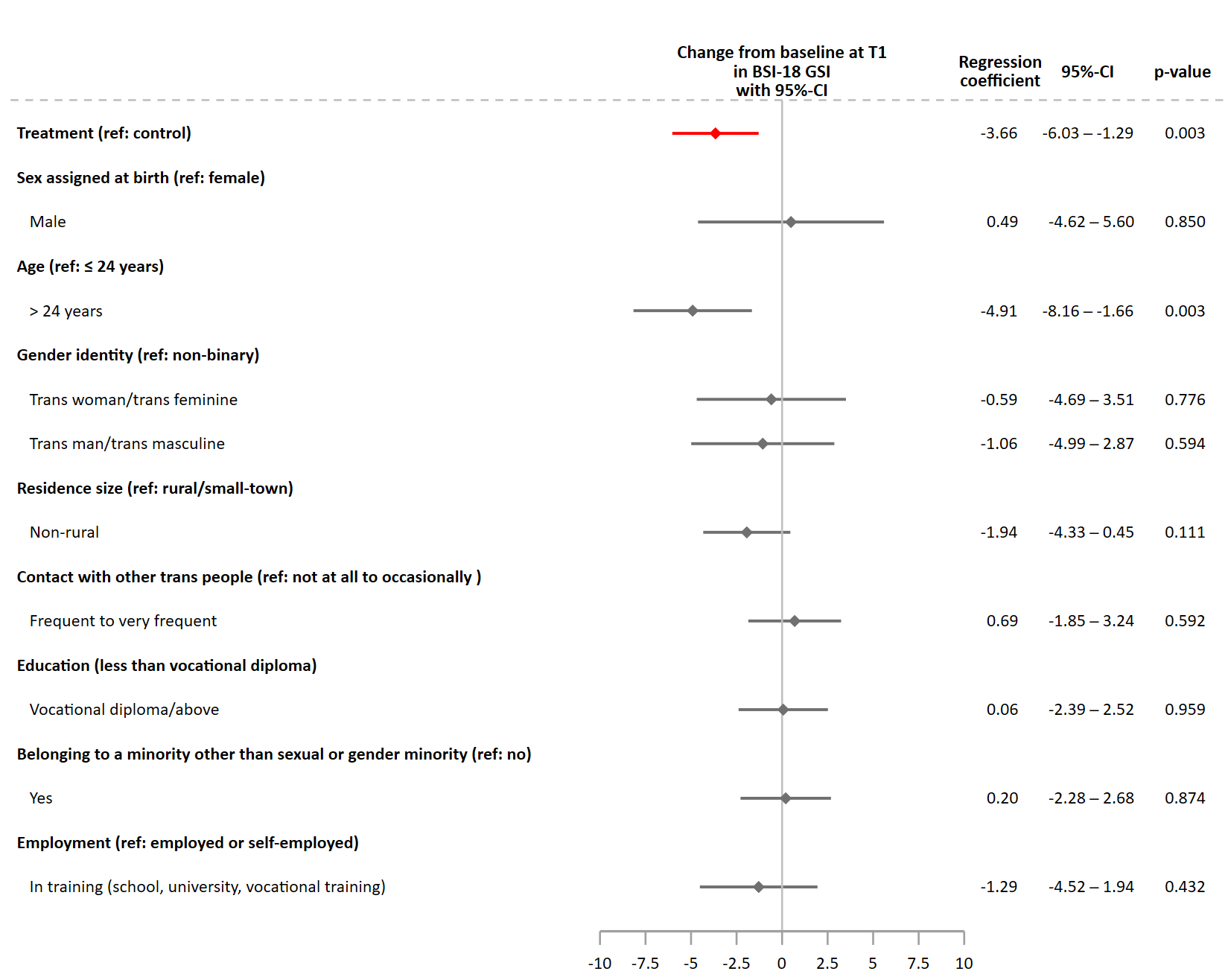

Supplement: Supplemental Material [file WIJT_A_2465710_SM2354.docx]
